# Chemical reprogramming culture for the expansion of salivary gland epithelial basal progenitor cells

**DOI:** 10.1186/s13287-025-04295-5

**Published:** 2025-04-18

**Authors:** Ye Jin Jeong, Yongpyo Hong, Yeo-Jun Yoon, Nam Suk Sim, Seung-Min Hong, Jae-Yol Lim

**Affiliations:** 1 Department of Otorhinolaryngology, Yonsei University College of Medicine, Seoul, Republic of Korea; 2 Severance Hospital, Yonsei University College of Medicine, Seoul, Republic of Korea; 3 Gangnam Severance Hospital, Yonsei University College of Medicine, 211 Eonju-ro, Gangnam-gu, Seoul, 06273 Republic of Korea

**Keywords:** Salivary gland, Chemical reprogramming culture, Basal progenitor cell, Epithelial stem cell, Cell therapy

## Abstract

**Background:**

Salivary gland (SG) hypofunction presents a significant clinical challenge with limited treatment options. SG epithelial cells offer a promising approach due to their intrinsic tissue specificity and regenerative potential. However, the lack of efficient culture methods has hindered their clinical use.

**Methods:**

This study presents a chemical reprogramming culture (CRC) system that utilizes a combination of three small molecules for the long-term two-dimensional culture of human SG epithelial progenitor cells. We characterized the cultured cells, measured their organoid-forming efficiencies, and assessed their differentiation potential. To evaluate the therapeutic efficacy of the SG basal progenitor cells (SG-BPCs), we administered them into a mouse model with radiation-induced SG hypofunction and assessed the functional recovery.

**Results:**

By utilizing optimal concentrations of the small molecules Y-27632, A83-01, and LDN193189, the SG epithelial cells achieved over 50 population doubling levels (PD) within 80 d, surpassing the Hayflick limit. β-galactosidase and Terminal deoxynucleotidyl transferase dUTP nick end labeling staining confirmed that these small molecules inhibited cellular senescence and apoptosis, respectively. The cells expressed SG basal ductal cell markers KRT5, KRT19, and SOX9, with increased expression levels observed from PD5 to PD40. Notably, these expanded cells were able to differentiate into various SG cell types, including acinar and myoepithelial cells, indicating that SG-basal progenitor cells (SG-BPCs) were selectively proliferated using our CRC method. To assess the therapeutic potential of the expanded SG-BPCs, they were administered to mice with radiation-induced SG hypofunction. The treatment successfully restored SG function.

**Conclusion:**

Our findings demonstrate that our CRC system is an effective method for the long-term culture of SG-BPCs. This advancement holds significant promise for the development of SG epithelial progenitor-based therapies to treat SG hypofunction.

**Supplementary Information:**

The online version contains supplementary material available at 10.1186/s13287-025-04295-5.

## Background

Numerous research groups have shown that the salivary gland (SG) tissue-resident stem cells can effectively restore SG hypofunction in mouse models of irradiation-induced or Sjögren’s syndrome-like conditions [1–3]. Despite these successful preclinical outcomes, clinical trials utilizing cell-based therapies to treat SG hypofunction have mainly focused on bone marrow-derived cells and mesenchymal stem cells (MSCs) [4, 5]. Although administered MSCs exhibit promising restoration of SG hypofunction, their paracrine functions may not be tissue-specific. SG-resident epithelial stem or progenitor cells (SG-EpSPCs) are expected to exhibit tissue-specific differentiation and paracrine secretion that targets SG cells. SG-EpSPCs have been cultured with animal-derived components and a cocktail of growth factors. By manipulating culture conditions, such as lowering calcium concentrations, SG cells have successfully induced differentiation into acinar-like cells [6]. Moreover, certain culture conditions have shown promise in maintaining the stability of SG cells up to passage 12, as evidenced by the expression of epithelial cell markers [7]. However, the use of animal-derived components and numerous growth factors in these culture systems poses significant limitations for clinical applications due to potential batch-to-batch variations, the risk of contamination, and transdifferentiation [8]. Additionally, these culture conditions, consisting of multiple cell types, complicate the isolation and expansion of specific cell populations of interest.

Obtaining high-quality SG-EpSPCs for clinical applications remains challenging for several reasons. First, human SG-EpSPCs cannot be ceaselessly maintained using a chemically defined epithelial cell culture medium in two-dimensional (2D) culture. Second, researchers have used three-dimensional (3D) cultures to overcome the limitations of 2D culture; however, 3D cultures are unable to isolate homogeneous stem cells. Finally, culturing 3D organoids is technically more challenging than 2D cultures as they consume more growth factors and utilize tumorigenic mouse-derived extracellular matrix resources. Although murine SG epithelial cells have been cultured under various conditions involving Transforming growth factor (TGF)-β inhibition and low Ca^2+^ concentrations [9], SG-EpSPCs are yet to be isolated and expanded under 2D culture conditions without feeder cells or matrices. Furthermore, a long-term 2D culture method for human SG-EpSPCs is yet to be developed.

A chemical reprogramming culture (CRC) was initially designed to maintain and proliferate epithelial cells over the long term by culturing cells with small molecules on irradiated J2 feeder cells [10]. However, the role of the J2 feeder in conditional reprogramming culture is not fully understood, and cell contamination still occurs, making its use in cell therapy challenging. A new technique was developed to grow epithelial cells of various organs using a biochemically defined medium by adding a TGF-β inhibitor, Bone morphogenetic protein (BMP) inhibitor, MEK1/2 inhibitor, or WNT agonist [11–13]. Somatic cell reprogramming offers a revolutionary approach to regenerative medicine by directly reprogramming mature cells into progenitor cells. This method bypasses the requirement for induced pluripotent stem cells (iPSCs), potentially reducing ethical concerns and immune rejection risks [14]. In this study, we demonstrate the generation of reprogrammed progenitor-like cells from epithelial cells. This was achieved through chemical reprogramming, a process that utilizes small molecule compounds to induce a transition to a progenitor-like state [15, 16]. Depending on the culture conditions, post-mitotic cells can acquire stem or progenitor cell characteristics, and terminally differentiated cells can maintain their function and proliferate during long-term culture. We developed a method to effectively proliferate human SG-basal progenitor cells (SG-BPCs) in 2D cell culture. The cultured cells showed epithelial morphology with at least 50 population doublings, indicating long-term culture stability. Furthermore, KRT5, KRT19, and SOX9 expression confirmed the maintenance of the ductal progenitor cell characteristics and their differentiation into acinar and myoepithelial cells. Notably, these cells can be administered or infused into the SG of mice to restore SG hypofunction. Cell-based therapies using SG-BPCs may hold great potential for treating SG dysfunction [17, 18]. The CRC method can be used to manufacture SG-BPCs under 2D conditions and provides a simple, cost-effective, and high-yield output of SG-BPCs.

## Materials and methods

### Human chemically reprogrammed SG epithelial progenitor cells isolation and expansion

Normal human parotid gland tissues were obtained from three patients with benign tumors. The patient demographics were as follows: average age: 30.0, gender: female (3/3), and tumor: benign (3/3). Human parotid gland tissue was cut into small pieces using a razor blade. The cut pieces were enzymatically digested with collagenase type II (#17101015; Thermo Fisher Scientific) for 1 h at 37 °C in a shaking incubator. After incubation, tissues were digested with TrypLE Express (#12604013; Thermo Fisher Scientific) and 10 μM Y-27632 for 10 min in a 37 °C bead bath. Cells were filtered through a 70-μm strainer and seeded in Keratinocyte serum-free-medium (KSFM; #37010022; Gibco) or DermaCult keratinocyte expansion medium (KEM; #100-0500; STEMCELL Technologies) with small molecules. The CRC media for culturing SG-EpSPCs contained DermaCult KEM supplemented with 0.1% Primocin, 10 μM Y-27632 (#1254; Tocris Bioscience), 1 μM TGF-β inhibitor A83-01 (#2939; Tocris Bioscience), BMP inhibitor 1 μM DMH-1 (#4126; Tocris Bioscience), or 0.1 μM LDN193189 (#6053; Tocris Bioscience). The medium was changed every 2–3 d. SG-EpSPCs were subcultured every 2–3 d. For passaging, the cells were washed with Dulbecco’s phosphate-buffered saline (PBS). The cells were dissociated with TrypLE Express for 10 min in a 37 °C bead bath. Dissociated cells were seeded in the culture media. For cryopreservation, dissociated cells from the parotid gland or cultured cells were mixed with cell-freezing media (#BLC-1; Zenoaq) supplemented with 10 μM Y-27632 and stored at − 80 °C in a deep freezer for 3 d and transferred to liquid nitrogen for long-term storage.

### Cumulative population doubling level and population doubling time

The cultured cells were counted using an automated cell counter (Countess™ 3 FL; Thermo Fisher Scientific) and the viability was determined using trypan blue staining. The cumulative population doubling level (PD) was calculated by substituting the cell counts obtained at each passage into the following formula [19]. Population doubling time (PDT) was calculated using the formula PDT = ln(Nt/N₀)/ln(2), where Nt is the cell number at time t, and N₀ is the initial cell number.

PD = log_2_(Nt/N₀)

Nt: Cell count at a specific time point t

N₀: Initial cell count

### Karyotype analysis

The cells were cultured in KEM or KEM supplemented with Y-27632, A83-01, and LDN193189 (KEM + YAL) for 15 passages. Karyotype analysis was conducted by GenDix, Inc. (92 Daehak-ro 12-gil, Jongno-gu, Seoul).

### Immunocytochemistry staining (ICC)

Cultured cells were rinsed three times with PBS for 5 min each and fixed using either 1:1 mixture of methanol and acetone at room temperature for 5 min or 4% paraformaldehyde for 10 min at room temperature. The cells were then rinsed with PBS and permeabilized with 0.3% Triton-X for 5 min at room temperature. Permeabilized cells were incubated with normal goat serum for 1 h at room temperature. The cells were incubated with primary antibody at 4 °C overnight. The following antibodies were used: KRT5 (1:1000; #905504; BioLegend), KRT7 (1:500; #sc-23876; Santa Cruz Biotechnology), KRT14 (1:500; #905304; BioLegend), AQP5 (1:200; #sc-514022; Santa Cruz Biotechnology), KRT19 (1:500; #ab52625; Abcam), Vimentin (1:1000; #ab8978; Abcam), E-cadherin (1:300; #3195S; CST), and ACTA2 (1:200; #ab7817; Abcam). The cells were then stained with secondary antibodies. Secondary antibodies were used at a 1:1000 dilution and the nuclei were counterstained with Hoechst 33342 (#R37605; Thermo Fisher Scientific). Data were analyzed using NIS-Elements BR (Nikon) or ZEN (Carl Zeiss) software.

### Quantitative real time-PCR (qRT-PCR)

Total RNA was extracted using the RNeasy Plus Mini kit (#74134; Qiagen) and reverse transcribed into cDNA using the PrimeScript RT reagent kit (#RR037; Takara) according to the instructions provided by the manufacturer. The qRT-PCR was performed using SYBR green dye and a QuantStudio 5 real-time PCR system (Thermo Fisher Scientific). The primers used are listed in Supplementary Table S1.

### Flow cytometry analysis

Chemically reprogrammed SG epithelial cells were filtered through a 35-μm nylon mesh (#352235; Corning). The cells were resuspended in PBS supplemented with 0.1% bovine serum albumin, 10 mM HEPES, and 25 mM EDTA. Single-cell suspensions were stained with Zombie violet (#423114; BioLegend) to eliminate dead cells and then incubated with human TruStain FcX (#422302; BioLegend). Thereafter, the single cells were stained with primary antibodies for 30 min at 4 °C. Stained cells were acquired using an LSRFortessa (BD Biosciences, Franklin Lakes, NJ, USA). Data were analyzed using FlowJo software. The antibodies used in this study are listed in Supplementary Table S2.

### β-galactosidase (β-gal) staining

To determine β-gal activity, a β-gal activity assay was performed following the kit protocol (#CS0030; Sigma). Briefly, the cells were fixed in 4% paraformaldehyde solution for 15 min at room temperature. After permeabilization with 0.1% Triton X-100 for 5 min, the cells were incubated with X-gal staining solution overnight at 37 °C. Blue-stained cells were quantified using a light microscope.

### Differentiation of SG stem cells

Single-cell suspensions derived from KEM and KEM + YAL samples were embedded in Matrigel and cultured in KEM or KEM + YAL media, respectively. Single cells were seeded at a density of 5 × 10^2^ cells in the Matrigel. The medium was replaced every 2–3 d, and the organoids were cultured for 9 d. Organoids were visualized using a phase-contrast microscope, and its formation efficiency was determined by enumerating the organoids per well. Organoids were defined as spherical structures with a diameter greater than 50 μm. The number of organoids per well was counted using the ImageJ software. Organoid formation efficiency was calculated as follows: (number of formed organoids/total number of seeded cells) × 100.

### Whole-mount staining

Organoids were fixed in 4% paraformaldehyde at room temperature for 15 min to preserve the cellular morphology and prevent autolysis. The fixed organoids were permeabilized with 0.1% Triton X-100 in PBS for 10 min at room temperature to allow the antibodies to penetrate the cells. Non-specific binding of antibodies was minimized by incubating the organoids in a blocking solution containing 5% normal goat serum in PBS for 1 h at room temperature. Organoids were then incubated with primary antibodies specific to the target proteins. The following antibodies were used: KRT5 (1:500; #905504; BioLegend), KRT7 (1:50; #sc-23876; Santa Cruz Biotechnology), AQP5 (1:200; #sc-514022; Santa Cruz Biotechnology), CDH1 (1:200; #3195S; CST), ACTA2 (1:200; #ab7817; Abcam), AMY1 (1:500; #PA5-78771; Invitrogen), MIST1 (1:250; #ab187978; Abcam) and TJP1 (1:50; #33–9100; Invitrogen). Secondary antibodies were used at a 1:1000 dilution. Nuclei were counterstained with Hoechst 33342 (#R37605; Thermo Fisher Scientific). Images were captured by confocal microscopy.

### Terminal deoxynucleotidyl transferase dUTP nick end labeling (TUNEL) assay

A TUNEL assay was performed to detect apoptotic cells following the manufacturer’s protocol (#S7100; Sigma). Briefly, the cells were fixed in 1% paraformaldehyde solution for 10 min at room temperature. After quenching with 3% hydrogen peroxide in PBS for 5 min, the cells were treated with an equilibration buffer for 10 s at room temperature. The cells were subjected to TdT enzyme for 1 h at 37 °C, followed by washing with stop/wash buffer for 10 min at room temperature. The cells were then treated with an anti-digoxigenin peroxidase conjugate for 30 min at room temperature and subsequently stained with a peroxidase substrate for 5 min at room temperature. Blue-colored cells were quantified using a fluorescence microscope.

### Western blotting

Whole cell lysates were subjected to 10% SDS-PAGE and transferred onto PVDF membranes. Membranes were blocked with 5% skim milk and incubated with primary antibodies against p21 (1:000; #2947; CST), p65 (1:1000; #6956; CST), p-p65 (1:500; #3033; CST), SOX2 (1:1000; #9795; Abcam), SOX9 (1:500; #82630; CST), ID3 (1:500; #9837; CST) and β-actin (1:3000; #4967; CST). After washing, membranes were incubated with HRP-conjugated anti-rabbit (1:5000; #31430; Invitrogen) or anti-mouse IgG secondary antibodies (1:5000; #7074; CST) and visualized using ECL solution.

### Gene expression and transcriptome analysis

For RNA sequencing, we used STAR align and Stringtie packages. The raw sequencing files were trimmed using Trimmomatic [20]. Trimmed sequencing data were aligned using STAR align against the hg38.refGene [21, 22]. Raw counts were assigned based on Ensembl human gene annotations using Stringtie [23]. The raw count and transcripts per million were calculated using Stringtie. Using the DEseq2 R package, the principal components and differentially expressed genes were analyzed [24]. Overall, changes in gene expression were considered significant if they passed the false discovery rate threshold of < 10%. Genes significantly affected by at least twofold were analyzed for enrichment. The batch effect was removed using limma packages [25]. Differentially expressed genes were analyzed using clusterprofiler [26]. The enrichment scores for each pathway were calculated using the GSVA R package [27].

### Mice and in vivo experiment

Seven-week-old female C57BL/6 mice were purchased from the OrientBio (Korea), fed ad libitum, and maintained under 22 ± 2 °C and 50 ± 10% relative humidity on a 12-h light/dark cycle (8 AM–8 PM) under specific pathogen-free conditions in a facility accredited by AAALAC International (#001071). The SG of the mice was locally irradiated with a single X-ray dose of 15 Gy (X-rad 320; Precision X-ray; North Branford, CT, USA). After 3 weeks, a total of 30 mice were randomly divided into groups for short-term and long-term effect evaluations. The short-term and long-term time point groups were as follows: non-irradiated and administered normal saline (non-IR group, n = 5), irradiated and administered normal saline (IR + Saline group, n = 5), and irradiated and administered 1 × 10^5^ SG-BPCs (IR + SG-BPCs group, n = 5). The sample size was decided according to the guidelines for sample size of Yonsei University. Subsequently, cells were administered through a cannula inserted in the submandibular duct orifice at a concentration of 1 × 10^5^ SG-BPCs/20 μL and a rate of 10 μL/min as previously described [28]. Mice injected with the same volume of normal saline served as controls. At 3 and 8 weeks post-SG-BPCs administration, mice were intraperitoneally administered 2 mg/kg pilocarpine (#P6503; Sigma) to stimulate salivary secretion under isoflurane anesthesia. Mice were subjected to general anesthesia with inhaled isoflurane using a small animal anesthesia machine (#34-1352; Harvard Apparatus). The mice were initially anesthetized rapidly within the pre-anesthesia chamber and then maintained at a 1.5–2% isoflurane concentration. We did not collect any samples from the animal that expired during the experimental procedure. Saliva content was measured using a cotton swab. The quantity of saliva was determined gravimetrically, assuming a density of 1 g/mL saliva. The mice were euthanized by carbon dioxide for 5 min, and the excised submandibular salivary glands (SMGs) were weighed and compared between the groups. The animal experiments have been reported in line with the ARRIVE guidelines 2.0.

### Tissue histology

The SMGs were fixed, embedded in paraffin, sectioned, and deparaffinized. The sections were stained with hematoxylin and eosin (HE; #ab245880; Abcam), periodic acid–Schiff (PAS; #ab150680; Abcam), and Masson’s trichrome (MTC; #ab150686; Abcam) according to the instructions provided by the manufacturer. Two blinded examiners assessed the pathological changes in HE, PAS, and MTC. SMG damage was quantified based on the morphological breakdown of the acinar and ductal structures in HE stained sections. Staining scores were recorded as previously described [29]. Scores between 0 and 5 were assigned based on the following criteria: intact acini in the image fields were scored “0” for ≥ 90%, “1” for 70–90%, “2” for 50–70%, “3” for 30–50%, “4” for 10–30%, and “5” for ≤ 10%. Mucin and fibrosis areas were measured by PAS and MTC staining, which indicated the area of mucin production as the ratio of magenta and the area of fibrosis as the blue color. The area was analyzed using ImageJ software, and the quantity of mucin and fibrosis areas was displayed as a percentage of the image fields.

### Immunofluorescence staining

Mouse SMG tissue samples were fixed in 4% paraformaldehyde for 24 h at 4 °C. Following fixation, tissues were embedded in paraffin and sectioned at 5 μm using a microtome. Sections were mounted on slides and de-paraffinized using xylene and graded alcohols. For antigen retrieval, slides were subjected to heat-induced epitope retrieval in Tris–EDTA buffer at 98.7 °C for 40 min. Slides were then blocked with 5% NDS in TBS for 1 h at room temperature. Subsequently, sections were incubated with primary antibodies against AQP5 (1:500; #AQP-005; Alomone) and KRT5 (1:1000; #905904; BioLegend) overnight at 4 °C. After washing, slides were incubated with Alexa Fluor 488-conjugated anti-rabbit (1:1000; #32790; Invitrogen) and Alexa Fluor 594-conjugated anti-chicken (1:1000; #703-585-155; Jackson ImmunoResearch) for 1 h at room temperature in the dark. Following additional washes with TBS, slides were mounted with Hoechst 33342 (#R37605; Thermo Fisher Scientific). Images were captured by confocal microscopy.

### Statistical analysis

To minimize bias, personnel conducting data collection and analysis were blinded to group assignments. A two-tailed independent t-test was used to compare the two groups. For more than two groups, a one-way analysis of variance (ANOVA) was conducted to compare the values and evaluate statistical significance. For three or more groups with an additional factor, a two-way ANOVA was conducted to assess the overall effect of each factor and their interaction on the outcome variable. Tukey’s post-hoc multiple comparison test was used to identify specific group differences following ANOVA. All statistical analyses were performed using GraphPad Prism 10 software (GraphPad Software, San Diego, CA, USA). The significance levels used in this study were * *p* < 0.05, ** *p* < 0.01, and *** *p* < 0.001.

## Results

### Small-molecule cocktails enable the long-term proliferation of SG epithelial cells

**Fig. 1:**
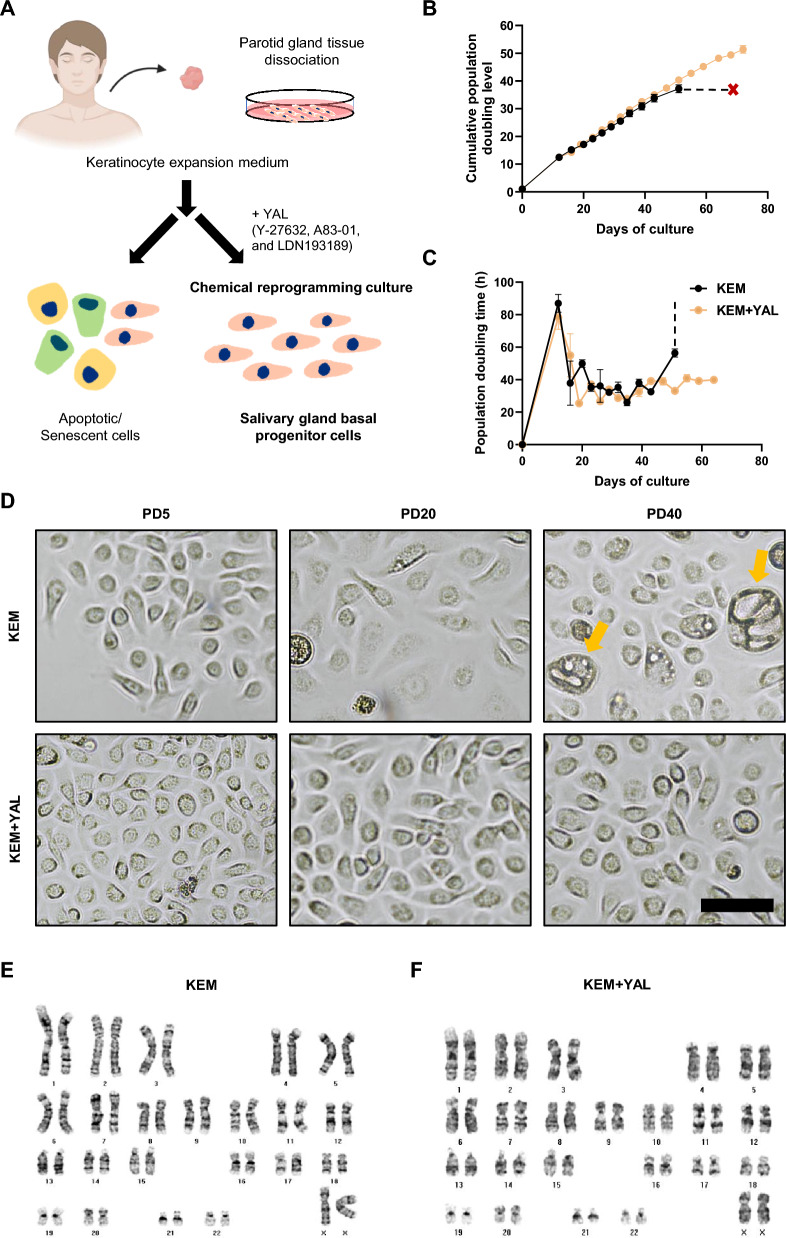
Small-molecule cocktails enables the long-term proliferation of SG epithelial cells. **A** Schematic depiction of CRC. **B** PD of human SG epithelial cells cultured with KEM in the absence and presence of a small-molecule cocktails. The data are representative of three independent experiments performed in triplicate and are expressed as mean ± SEM. **C** PDT of KEM and KEM + YAL conditions. The data are representative of three independent experiments performed in triplicate and are expressed as mean ± SEM. **D** Representative images of SG epithelial cells cultured under KEM and KEM + YAL conditions at PD5, PD20, and PD40 time points. The PD40 cells under KEM condition exhibit a senescent phenotype (yellow arrow). Scale bar: 100 μm. **E**, **F** Karyotype analysis is conducted with SG epithelial cells at PD40 under KEM and KEM + YAL conditions, respectively

First, we explored various small-molecule-conditioned epithelial cell cultures of human epithelial stem or progenitor cells [30, 31]. Inhibition of BMP and TGF-β signaling pathways through small-molecule-based culture methods can reportedly promote the proliferation of basal progenitor cells and suppress their differentiation [32, 33]. Furthermore, a Rho kinase (ROCK) inhibitor has been shown to delay senescence in stem cells [34, 35]. Based on these findings, we hypothesized that a combined approach utilizing BMP and TGF-β inhibitors, along with a ROCK inhibitor, could enhance the proliferation of basal progenitor cell populations within the SG. After obtaining human SG cells, we screened several small-molecule combinations and found that some small molecules, including Y-27632, which is a ROCK inhibitor; A83-01, which is a TGF-β inhibitor; and DMH-1, which is a BMP inhibitor, achieved a population doubling 50 approximately 2 times faster than an epithelial cell culture without small molecules in KEM. Additionally, the cells cultured in the triple combination of 10 µM Y-27632, 1 µM A83-01, and 1 µM DMH-1(YAD) reached population doubling 50 approximately 1.5-times faster compared to the cells cultured in the dual combination of 10 µM Y-27632 and 1 µM A83-01 (YA) (Fig. S1A). The cultured cells exhibited a cobblestone appearance, typical of epithelial cells (Fig. S1B). In addition, DMH-1, a selective BMP inhibitor for the ALK2 receptor, inhibits autophagy and may have long-term negative effects on the cells [36]; hence, subsequent experiments were conducted using LDN193189, an alternative BMP inhibitor for ALK2, ALK3, and ALK6 receptors [37]. Based on this triple chemical combination, we then tested the impact of small-molecule combinations on SG epithelial cell cultures (Fig. 1A). To determine the optimal combination of small-molecule concentrations, we cultured the cells with varying concentrations of each small molecule. First, the TGF-β inhibitor A83-01 showed the highest population doublings of epithelial cells at concentrations of 0.2, 1, and 2 µM, compared to 0 and 0.5 µM (Fig. S1C). Similarly, the BMP inhibitor LDN193189 demonstrated the highest population doublings at 0.1 µM, whereas the ROCK inhibitor Y-27632 showed the highest population doublings at 10.0 µM (Fig. S1D and E). SG epithelial cells cultured with optimal concentrations of the small molecules Y-27632, A83-01, and LDN193189 in KEM underwent over 50 divisions within 80 d, surpassing the Hayflick limit. In contrast, cells cultured in KEM without small molecules underwent fewer than 40 divisions and could not be maintained beyond 60 d (Fig. 1B). Additionally, the PDT of epithelial cells cultured in KEM + YAL was stabilized with repeated passaging, whereas that of cells cultured in KEM showed variability and was prolonged after 40 d, leading to an inability to continue culturing (Fig. 1C). On PD5 and PD20, cells cultured in KEM + YAL appeared smaller and more densely packed than those cultured in KEM alone. At PD40, the maximum passage level for SG epithelial cells in KEM had been achieved. A comparison with cells cultured in KEM + YAL using high-magnification bright-field microscopy revealed that the cells were enlarged and appeared flattened under a better focus, suggesting that cellular aging had occurred (Fig. 1D). In vitro passage cultures may induce genomic instability. To address this, we performed karyotyping at maximum passage levels in both KEM and KEM + YAL. No chromosomal breakage or alterations were observed under either culture condition (Fig. 1E and F). Overall, we established a basal medium combined with small molecules that allowed for long-term culture of SG epithelial cells in vitro without inducing genomic instability.

### A triple small-molecule cocktail preferentially enabled the expansion of SG basal ductal cells

**Fig. 2:**
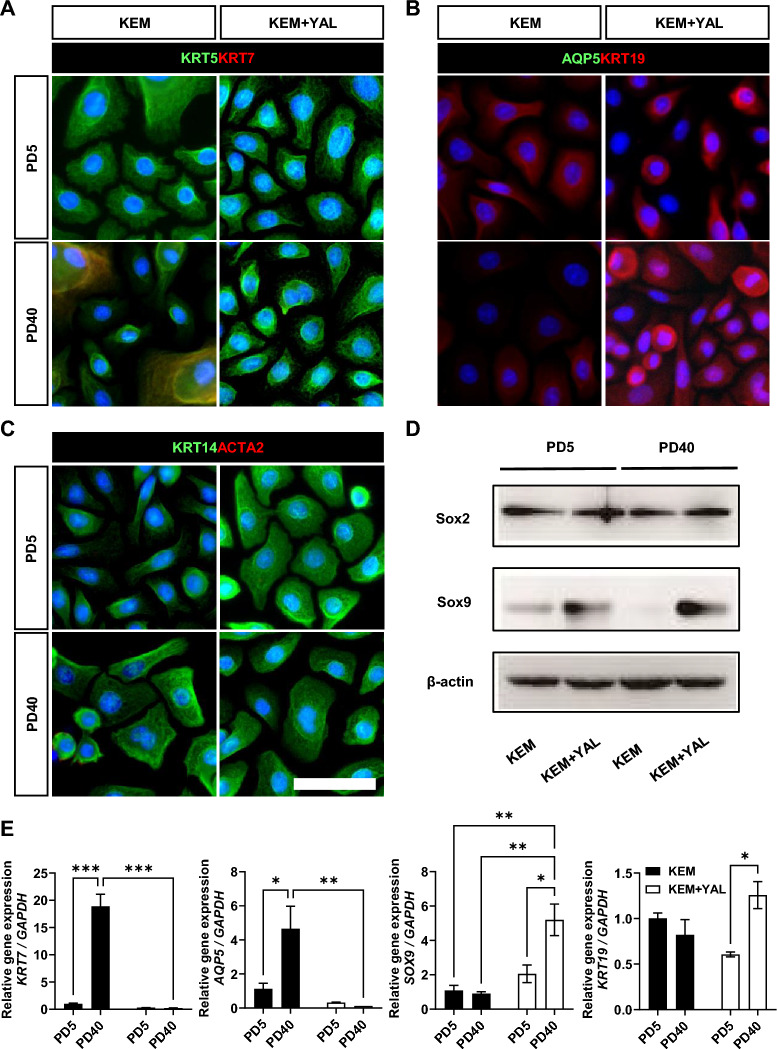
Triple small-molecule cocktail preferentially enables the expansion of SG basal ductal cells. **A** Representative ICC staining for KRT5 (basal cell, green), KRT7 (terminally differentiated luminal cell, red); **B** AQP5 (pro-acinar cell, green), KRT19 (ductal progenitor cell, red); and **C** KRT14 (ductal progenitor cell, green), ACTA2 (myoepithelial cell, red), and DAPI (blue) of human SG epithelial cells under KEM and KEM + YAL conditions at PD5 and PD40. Scale bar: 50 μm. **D** Western blot of the cells cultured under KEM and KEM + YAL conditions at PD5 and PD40 for SOX2 (acinar progenitor cell) and SOX9 (ductal progenitor cell). Full-length blots are presented in Fig. S7A. **E** mRNA expression of *KRT7*, *AQP5*, *SOX9*, and *KRT19* in cells cultured under KEM and KEM + YAL conditions, measured by qRT-PCR. Two-way ANOVA (alpha = 0.05) is conducted on data presented in (**E**) followed by Tukey’s multiple comparisons. The data are representative of three independent experiments performed in triplicate and are expressed as mean ± SEM. * = *p* < 0.05, ** = *p* < 0.01, *** = *p* < 0.001

Next, we investigated the changes in cell type upon long-term culture in KEM and KEM + YAL. We performed co-immunofluorescence staining using various antibodies against cells cultured on PD5 and PD40, in both KEM and KEM + YAL. KRT5, a marker of salivary basal ductal progenitor cells, and KRT7, a marker of fully differentiated luminal ductal cells, were analyzed. At PD5, both culture conditions strongly expressed KRT5; however, KRT7 expression was not detected. At PD40, KRT7 expression was observed in KEM but not in KEM + YAL (Fig. 2A). We also conducted co-immunofluorescence staining for AQP5, a marker for pro-acinar cells, and KRT19, which is suspected to be a marker for ductal progenitor cells. AQP5 expression was not observed at PD40 under either culture conditions. KRT19 was enriched in the KEM + YAL culture at PD40, whereas it was reduced in KEM, suggesting that KEM conditions do not support cell self-renewal (Fig. 2B). As myoepithelial cells have also been studied as reserved stem or progenitor cells in various exocrine glands, including the SG [38, 39], we verified whether the cultured cells included myoepithelial cells by performing co-immunofluorescence staining for KRT14, a basal and myoepithelial cell marker, and ACTA2, a myoepithelial cell marker. We did not detect ACTA2 expression under either KEM or KEM + YAL culture conditions. However, the distinct expression of KRT14 confirmed the presence of KRT14 + ACTA2- basal cells (Fig. 2C). Additionally, we assessed the expression of the acinar cell progenitor marker SOX2 and ductal cell progenitor marker SOX9 by western blotting. We detected SOX2 expression in both KEM and KEM + YAL cultures at PD5 and PD40. SOX9 expression was observed in KEM culture at PD5 but not at PD40, whereas under KEM + YAL conditions, SOX9 expression increased from PD5 to PD40 (Fig. 2D). mRNA expression analysis revealed that the levels of gene expression of *KRT7* and *AQP5* increased over time in KEM but were low and remained nearly unchanged in KEM + YAL. In contrast, ductal progenitor-related genes, such as *SOX9* and *KRT19*, exhibited a decreasing trend in KEM-cultured cells but were enriched significantly with passage in cells cultured in KEM + YAL (Fig. 2E). The expression of Yamanaka factors is currently being studied as a part of rejuvenation medicine. Partial Yamanaka factor expression can maintain the original phenotype and function of the cell [40]. Therefore, we investigated whether an increase in Yamanaka factor expression influences the expansion of SG epithelial cells in vitro. Cells cultured in KEM + YAL showed either statistically insignificant increases or no increase in *POU5F1*, *KLF4*, *SOX2*, and *c-MYC* expression at both PD5 and PD40. In contrast, KEM-cultured cells exhibited a significant increase in the Yamanaka factors, except *c-MYC* (Fig. S2A). The epithelial cell marker gene, *CDH1*, increased in KEM, whereas the transiently expressed cell marker gene [41], *VIM,* experienced a greater increase under KEM + YAL conditions than under the control conditions of KEM. The increased expression of *VIM* in KEM + YAL-cultured cells suggested that these conditions sustained cell expansion [42] (Fig. S2B). Immunofluorescence staining revealed the expression of Vimentin was not observed, whereas E-Cadherin, an epithelial cell marker, was observed between cells (Fig. S2C). These results suggest that KEM + YAL selectively expands salivary basal ductal cells over time.

### Stem cell characteristics and differentiation potential of chemically reprogrammed SG epithelial cells with triple small molecules

**Fig. 3:**
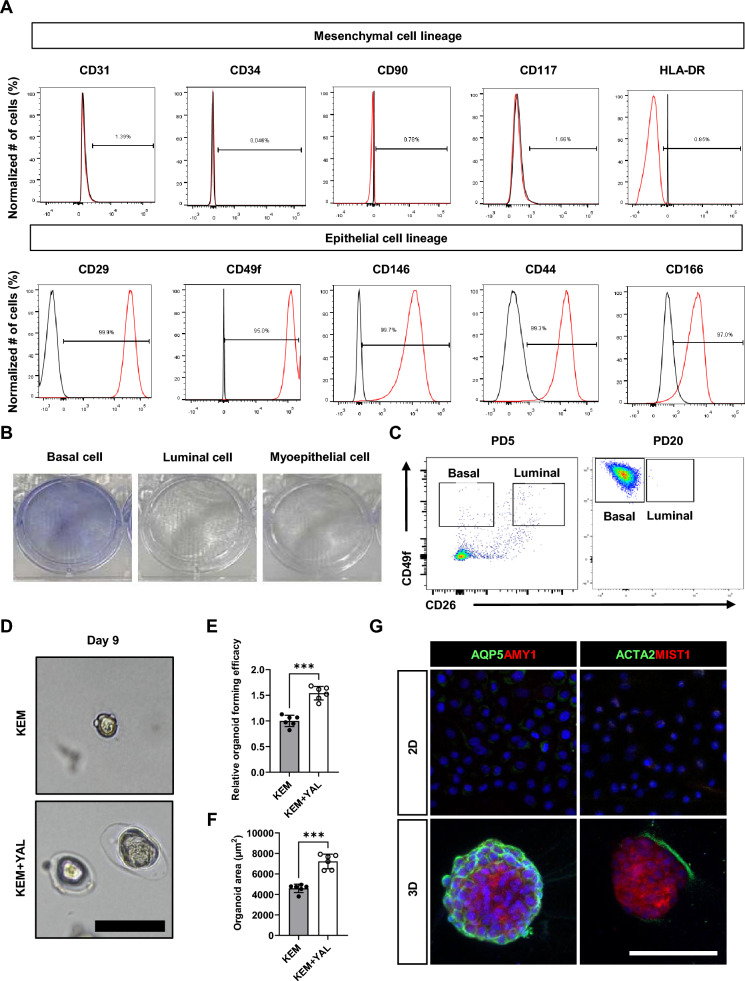
Stem-cell characteristics and differentiation potential of chemically reprogrammed SG epithelial cells with triple small molecules. **A** Flow cytometry is performed to assess the expression of various cell-surface markers in KEM + YAL-cultured PD20 cells (red line). The SG epithelial cells are negative for CD31, CD34, CD90, CD117, and HLA-DR, whereas they are positive for CD29, CD49f, CD146, CD44, and CD166. Isotype controls identify negative controls (black line). **B** The KEM + YAL-cultured cells are isolated using fluorescence-activated cell sorting and crystal violet staining of basal, luminal, and myoepithelial cells. **C** Flow cytometric analysis reveals an increase in the proportion of basal cells in PD20 cells compared to that in PD5 cells. **D** SG epithelial cells from KEM and KEM + YAL conditions are cultured in Matrigel for 9 d. Identical fields are captured using a bright field microscope. Scale bar: 100 μm. **E** Quantification of organoid forming efficacy. **F** Quantification of organoid area. An unpaired Two-tailed t-test is used to calculate significance on data presented in (**E**) and (**F**). Quantification is conducted from a single experiment with three technical repeats and expressed as mean ± SD. *** = *p* < 0.001. **G** Immunofluorescence microscopy of AQP5, AMY1, ACTA2, and MIST1 on both 2D and 3D cultures for 9 d. Scale bar: 100 μm

We sought to characterize SG epithelial cells cultured under KEM + YAL conditions and identify their stem cell properties. Flow cytometric analysis of KEM + YAL-cultured cells using various antibodies revealed that markers for mesenchymal and hematopoietic or endothelial stem cells, such as CD31, CD34, CD90, CD117, and HLA-DR, were negative. In contrast, cells were positive for markers associated with SG epithelial stem cells, including CD29, CD49f, CD146, CD44, and CD166 (Fig. 3A). We seeded 1,000 individuals basal, luminal/acinar, and myoepithelial cells in wells that could be sorted by CD26 and CD49f, based on our previous research [43]. After sorting, each cell line was cultured under CRC conditions for 5 d. Colony formation was observed only in basal cell-seeded wells, and no colonies were found in luminal/acinar or myoepithelial cell cultures. After 2 weeks, we performed the MTT assay and observed purple formazan crystals only in the basal cell culture wells, suggesting that acinar, luminal, and myoepithelial cells could not grow under our CRC conditions (Fig. 3B). Flow cytometric analysis using CD49f and CD26 markers was conducted to characterize the cellular populations following treatment with small-molecule cocktails. Although no notable differences were observed in the ratio of CD49^+^CD26^−^ basal and CD49f^+^CD26^+^ luminal cells at PD5, a significant shift toward a basal cell phenotype was observed at PD20, with more than 95% of the cells expressing the CD49f^+^CD26^−^ basal cell marker (Fig. 3C). To verify whether SG epithelial cells possess stem cell potency, we assessed their organoid formation capability and the presence of differentiated cells in 3D culture. The organoids were cultured in Matrigel for 9 d using KEM or KEM + YAL. Organoids derived from KEM + YAL-cultured SG epithelial cells showed a higher capacity for organoid formation than those derived from KEM-cultured cells after 9 d (Fig. 3D and E). Furthermore, the area of organoids derived from KEM + YAL was significantly larger than that of KEM-derived organoids (Fig. 3F). These observations suggested that KEM + YAL conditions support the maintenance of stem-like cell characteristics and enable self-assembly in 3D cultures. After 9 d of culture, 2D- and 3D-cultured cells in KEM + YAL were analyzed by qRT-PCR and IF staining. IF staining showed increased expression of AQP5; AMY1; MIST1, a mature acinar cell marker; and CDH1, whereas KRT5 expression remained unchanged. Notably, the myoepithelial cell marker (ACTA2) was expressed in 3D cultures. However, KRT7 and tight-junction marker (TJP1) were not detected in 3D cultures (Figs. 3G and S3A). qRT-PCR results showed that 3D cultured cells had elevated expression of the luminal ductal cell marker gene (*KRT7*). The expression levels of ductal cell marker genes *KRT5*, *KRT14*, and *KRT19* remained unchanged. Moreover, the pro-acinar cell marker gene (*AQP5*) and parotid gland secretory marker genes (*AMY1A*, *BPIFA2*, and *MUC5B*) were upregulated (Fig. S3B). These results suggested that SG basal ductal cells cultured with small-molecule combinations exhibited an epithelial stem cell phenotype and possessed the potential to differentiate into salivary epithelial and myoepithelial cells. Collectively, our results demonstrated that chemically reprogrammed cultured cells exhibit features of SG-BPCs.

### Small molecules inhibited cellular senescence and apoptosis in SG-BPCs

**Fig. 4:**
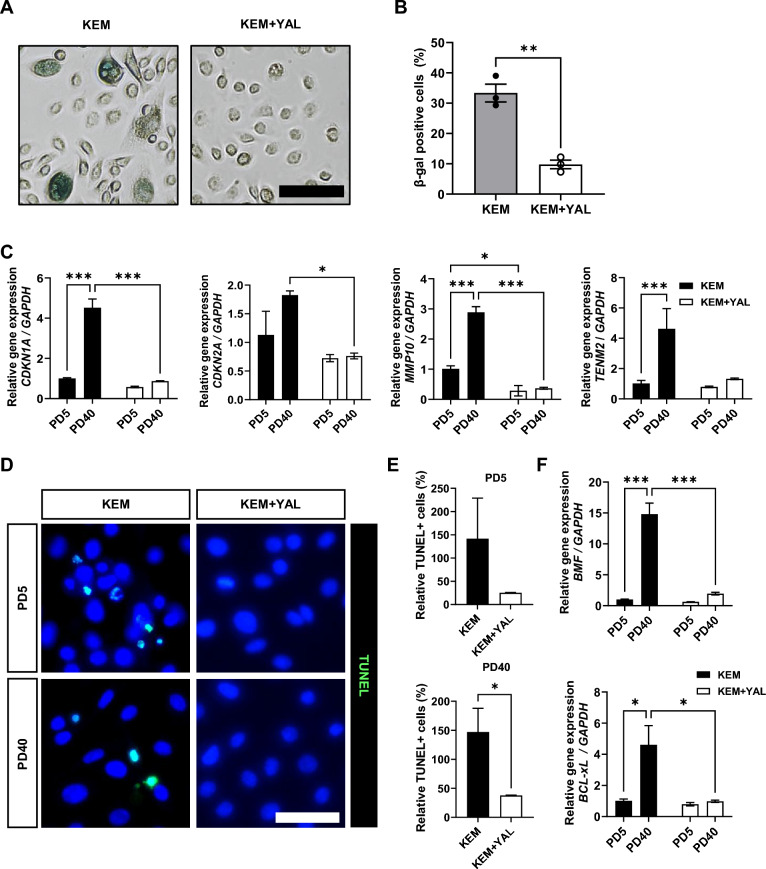
Small molecules inhibit activation of oncogene-induced senescence and apoptosis in SG-BPCs. **A** Representative SA-β-galactosidase staining images of SG epithelial cells cultured under KEM and KEM + YAL conditions at PD40. Scale bar: 100 μm. **B** The β-gal + cells are quantified in conditions without and with the small-molecule compounds. An unpaired Two-tailed t-test is used to calculate significance. Quantification is conducted from a single experiment with three technical repeats and expressed as mean ± SD. ** = *p* < 0.01. **C** The gene expression of cellular senescence marker (*CDKN1A*, *CDKN2A*, *MMP10*, and *TENM2*) in SG epithelial cells untreated and treated with small molecules. **D** Representative TUNEL staining images of SG epithelial cells in the KEM and KEM + YAL groups at PD5 and PD40. Scale bar: 50 μm. **E** TUNEL + cells are quantified in conditions without and with the small-molecule compounds. An unpaired Two-tailed t-test is used to calculate significance. Quantification is conducted from a single experiment with three technical repeats and expressed as mean ± SD. * = *p* < 0.05. **F** The gene expression of cellular apoptosis markers (*BMF* and *BCL-xL*) in SG epithelial cells untreated and treated with small molecules. Two-way ANOVA (alpha = 0.05) is conducted on data presented in (**C**) and (**F**) followed by Tukey’s multiple comparisons. The data are representative of three independent experiments performed in triplicate and are expressed as mean ± SEM. * = *p* < 0.05, *** = *p* < 0.001

We investigated whether the long-term proliferation and maintenance of epithelial cell properties cultured in KEM + YAL were associated with the inhibition of cellular senescence or cell death. β-gal staining, a standard method for assessing cellular senescence, revealed that approximately 30% of KEM-cultured cells were β-gal + , whereas < 10% of KEM + YAL-cultured cells were β-gal + (Fig. 4A and B). The protein expression of p21, a key regulator of the cell cycle, indicated its potential role in cell cycle arrest associated with senescence in KEM-cultured cells. However, the small-molecule cocktails did not induce appreciable changes in p21 protein levels (Fig. S4A). In addition, the expression of senescence-related genes such as *CDKN1A*, *CDKN2A*, *MMP10*, and *TENM2* increased progressively with passage number in KEM-cultured cells. In contrast, cells cultured in KEM + YAL exhibited no significant increase in the expression of these genes (Fig. 4C). We also examined whether these small molecules suppress apoptosis. TUNEL + cells were observed in SG epithelial cells cultured under KEM conditions, even at PD5, whereas no TUNEL + cells were detected in KEM + YAL-cultured cells, even at PD40 (Fig. 4D and E). Furthermore, apoptosis-related genes, such as *BMF* and *BCL-xL*, showed a significant increase in cells cultured in KEM, whereas their expression either showed a minimal increase or remained unchanged in cells cultured in KEM + YAL (Fig. 4F). To characterize the growth factor and cytokine profiles of cells treated with or without the small-molecule cocktails, we performed cytokine array analysis. CXCL8, IL-6, IL-15, IL-33, CCL19, TNFSF12, TSLP, CXCL9, CCL13, and CCL11 levels were significantly increased in cells cultured in small molecules compared to those in KEM conditions (Fig. S4B). Collectively, these results suggest that the added small molecules inhibit cellular senescence and apoptosis in SG epithelial cells, thereby enabling long-term culture in vitro.

### NF-κB signaling pathway supports the long-term maintenance of SG-BPCs

**Fig. 5:**
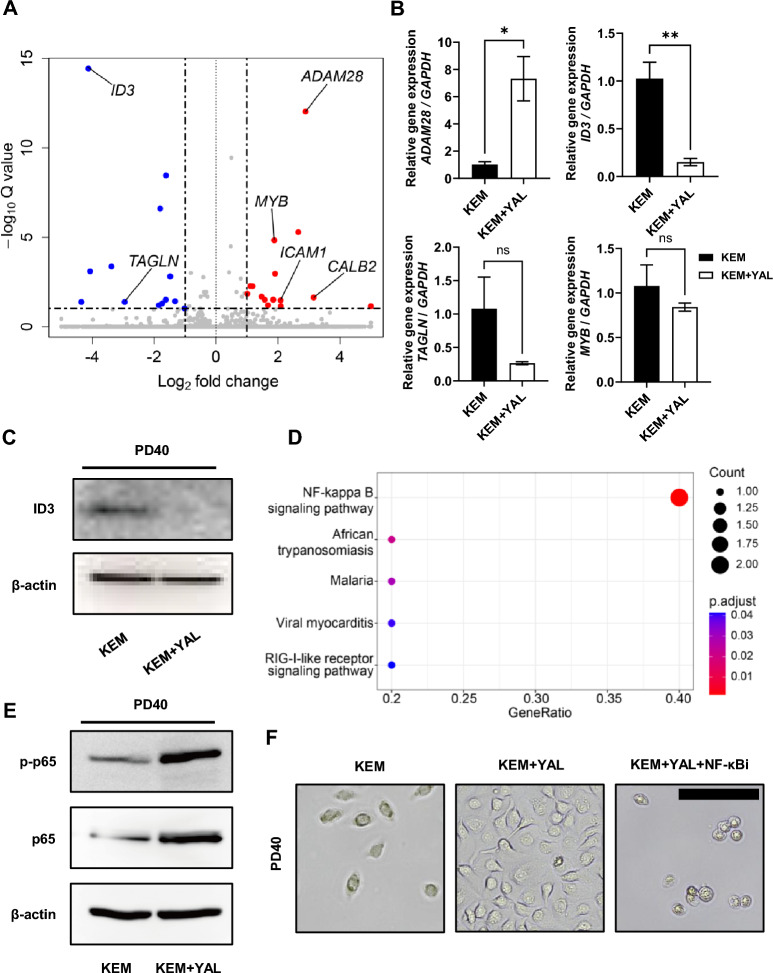
NF-κB signaling pathway supports the long-term maintenance of SG-BPCs. **A** Volcano plot depicting differential gene expression between KEM and KEM + YAL, as analyzed by bulk RNA sequencing. Data points are colored based on their average expression across all datasets. Red points represent genes upregulated in KEM + YAL, whereas blue points indicate downregulated genes. An adjusted *p* < 0.1 is considered significant for differential expression. **B** The gene expression of cellular senescence markers (*ADAM28, ID3, TAGLN,* and *MYB*) in SG-BPCs untreated and treated with small molecules. **C** Western blot of the cells cultured under KEM and KEM + YAL conditions at PD40 for ID3 and β-actin. Full-length blots are presented in Fig. S7C. **D** Dot plots illustrate the results of GSEA with an adjusted *p* < 0.1. Each dot plot highlights enriched terms within the transcriptome of KEM + YAL. The size of the dot corresponds to the number of enriched genes, and the color indicates the adjusted *p*-value. **E** Western blotting is performed to analyze protein expression levels of p-p65, p65, and β-actin in PD40 cells cultured without or with small-molecule cocktails. Blotting for p-p65, p65, and β-actin are performed sequentially on the same membrane. Full-length blots are presented in Fig. S7D. **F** Representative images of the cells cultured under KEM, KEM + YAL, and KEM + YAL + NF-κBi conditions for 72 h at PD40 time-point. The data are representative of three independent experiments performed in triplicate and are expressed as mean ± SEM. ns = not significant, * = *p* < 0.05, ** = *p* < 0.01

Bulk RNA sequencing was performed to investigate the mechanisms underlying the regulation of cellular senescence and apoptosis by the added small molecules. This analysis compared the gene expression profiles of cells cultured with KEM + YAL and those cultured with KEM. Volcano plot analysis at PD40 revealed that 38 genes were significantly differentially expressed (adjusted p < 0.05, |log2 fold change|> 1) (Fig. 5A). Significant upregulation of genes involved in cell adhesion and signaling, such as *Calbindin 2* (*CALB2*), *A Disintegrin And Metalloproteinase 28* (*ADAM28*), and *Intercellular Adhesion Molecule 1* (*ICAM1*), may enhance cell–cell interactions. Moreover, upregulation of the transcription factor *Myeloblastosis oncogene* (*MYB*) indicates increased cell proliferation. The downregulation of *Transgelin* (*TAGLN*) and *Inhibitor of DNA binding 3* (*ID3*), genes associated with the cytoskeleton and cell-cycle regulation, respectively, may contribute to the observed changes in cellular morphology. *ADAM28* exhibited the most significant upregulation among these genes, suggesting a potential role in cell proliferation [44]. Previous studies have reported the expression of *ADAM28* in the epithelial cells of various normal organs [45]. These findings suggest that *ADAM28* plays a protective role against C1q-mediated cell death in the bronchial epithelium, indicating a potential mechanism for maintaining epithelial integrity. *ID3* experienced the most significant downregulation among these genes. To validate the transcriptome analysis, we performed an mRNA expression analysis. Validation experiments revealed that *ADAM28* was upregulated in cells cultured with KEM + YAL, and *ID3* was downregulated in cells cultured with KEM. There was no significant difference between *TAGLN* and *MYB* under either culture conditions (Fig. 5B). Western blot analysis revealed increased ID3 protein expression in cells cultured in KEM (Fig. 5C). Gene Set Enrichment Analysis revealed that cells cultured under KEM + YAL conditions were enriched for hallmark signatures such as NF-κB signaling pathway and RIG-like receptor signaling pathway compared to KEM-cultured cells (Fig. 5D). To verify NF-κB activation, western blot analysis revealed increased levels of p-p65 and p65 in PD40 cells cultured with KEM + YAL (Fig. 5E). The NF-κB pathway is involved in DNA transcription, cytokine production, and cell survival, and is known to promote cell survival in the SG under conditions such as radiation exposure and obstructive sialadenitis [46, 47]. To further investigate the role of NF-κB in cell survival and proliferation, we treated KEM + YAL-cultured PD40 cells with the specific NF-κB inhibitor, BAY 11-7082. The cells were then incubated for 24, 48, or 72 h. At 24 and 48 h, the cells treated with the inhibitor showed a slight decrease in proliferation (Fig. S1A). When compared to the cells cultured with KEM + YAL, the BAY 11–7082-treated cells at 72 h were characterized by an enlarged cytoplasm and reduced colony formation, similar to the cells cultured with KEM. These results suggest that NF-κB inhibition by BAY 11-7082 reduces cell survival and proliferation (Fig. 5F). These findings indicate that small molecules promote a stable in vitro culture of SG-BPCs by activating the NF-κB pathway.

### Efficacy of SG-BPCs on tissue regeneration in a mouse radiation model

**Fig. 6:**
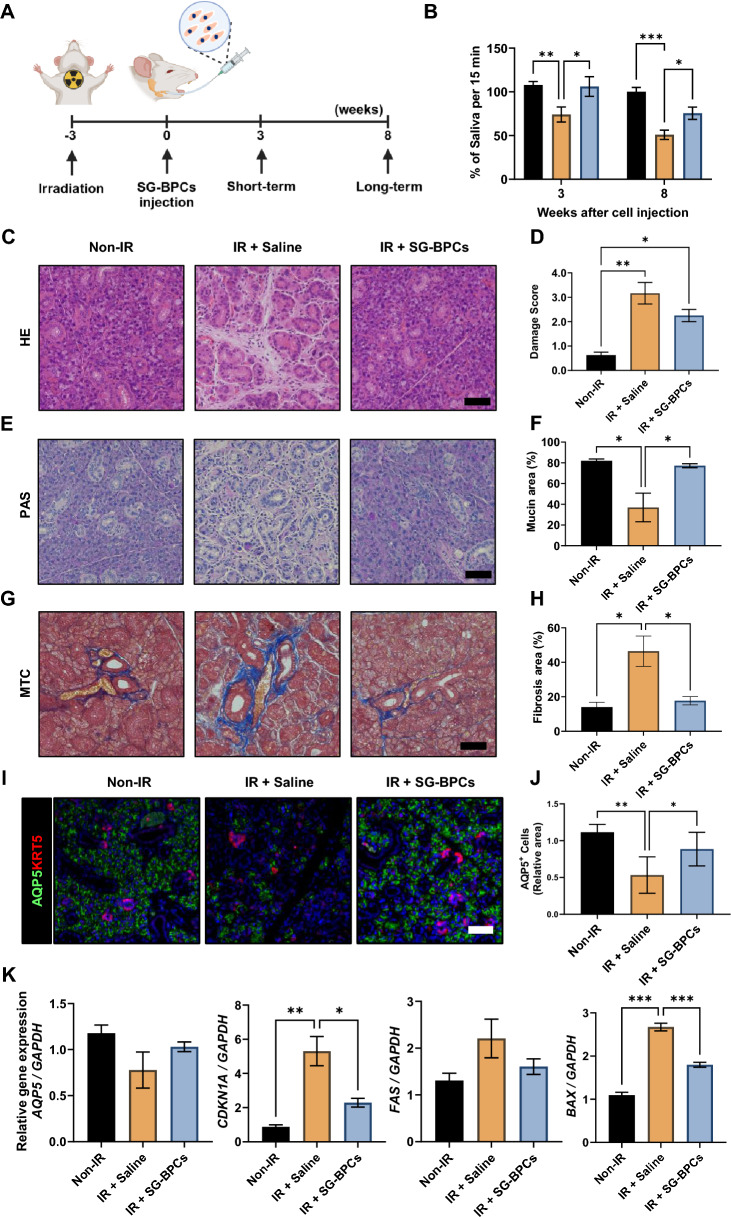
Efficacy of SG-BPCs on tissue regeneration in a mouse radiation model. **A** Schematic depiction of mouse experiment. **B** Relative saliva flow rate after 3 and 8 weeks of SG-BPCs injection. Two-way ANOVA (alpha = 0.05) is conducted on data presented in (**B**) followed by Tukey’s multiple comparisons. The data are representative of three independent experiments performed in triplicate and are expressed as mean ± SEM. * = *p* < 0.05, ** = *p* < 0.01, *** = *p* < 0.001. **C**, **E**, and **G** Representative histological images of HE, PAS, and MTC staining at Week 8 after SG-BPCs injection, respectively. Scale bar: 50 μm. **D**, **F** and **H** Quantification of SG damage score, ratio of mucin area, and fibrosis area, respectively. Two-way ANOVA (alpha = 0.05) is conducted on data presented in (**D**), (**F**), and (**H**) followed by Tukey’s multiple comparisons. The data are representative of three independent experiments performed in triplicate and are expressed as mean ± SEM. * = *p* < 0.05, ** = *p* < 0.01. **I** Immunofluorescence microscopy of AQP5 and KRT5 at 8 weeks after SG-BPCs injection. Scale bar: 50 μm. **J** Quantification of AQP5^+^ cells. One-way ANOVA (alpha = 0.05) is conducted on data presented in (**I**) followed by Tukey’s multiple comparisons. The data are representative of three independent experiments performed in triplicate and are expressed as mean ± SEM. * = *p* < 0.05, ** = *p* < 0.01. **K** Gene expression analysis of markers associated with acinar cells, cellular senescence, and apoptosis (*AQP5*, *CDKN1A*, *FAS,* and *BAX*). An unpaired two-tailed t-test is used to calculate significance. The data are representative of three independent experiments performed in triplicate and are expressed as mean ± SEM. * = *p* < 0.05, ** = *p* < 0.01, *** = *p* < 0.001

To investigate their potential for tissue regeneration in vivo, SG-BPCs were administered to the SMGs duct of irradiated mice. SMGs of C57BL/6 mice were locally irradiated with a single 15 Gy dose. After 3 weeks of irradiation, SG-BPCs were injected via the retroductal route (Fig. 6A). Saliva flow in the SG-BPCs-injected group increased significantly by week 3 and 8 after SG-BPCs injection compared with that in the saline-injected group (Fig. 6B). Furthermore, the weight of the SMGs increased significantly in the SG-BPCs-injected compared to that in the saline-injected group at 8 week (Fig. S6A). We conducted tissue staining to analyze the histology 8 weeks after SG-BPCs injection. Although not statistically significant, a decreasing trend in tissue damage, as measured by acinar atrophy and ductal dilation through HE staining, was observed in the SG-BPCs-injected compared to that in the saline-injected group (Fig. 6C and D). A significant increase in the mucin area ratio, as determined by PAS, was observed in the SG-BPCs-injected compared to the saline-injected group (Fig. 6E and F). Additionally, MTC staining showed a significant reduction in the fibrotic area compared to the saline-injected group (Fig. 6G and H). Immunofluorescence staining was performed to visualize AQP5 and KRT5 and confirm cellular reconstruction by the injected SG-BPCs. AQP5 expression was significantly higher in the SG-BPCs-injected group compared to the saline-injected group, while KRT5 expression was not statistically significant between groups (Figs . 6I, J, and S6B). To assess the regenerative effects of the injected SG-BPCs, we examined the expression of *AQP5*, a pro-acinar cell marker. Although not statistically significant, a trend toward increased *AQP5* expression was observed in the SG-BPCs-injected compared to that in the saline-injected group. Additionally, gene expression analysis of cellular senescence- and apoptosis-associated markers, such as *CDKN1A*, *FAS*, *BAX*, *BCL2*, and *CASP3*, revealed a significant decrease or decreasing trend in the SG-BPCs-injected group (Figs. 6J and S6C). These results show that SG-BPCs facilitate the recovery of SG structural regeneration, mucin production, and anti-fibrotic effects. The SG-BPCs-injected group showed no difference in SMG weight compared with the saline-injected group at week 3 (Fig. S6D). Furthermore, we conducted tissue staining to analyze the histology 3 weeks after SG-BPCs injection. The SG-BPCs-injected group had a reduction in tissue damage, as evident from the decreased acinar atrophy and ductal dilation observed in HE-stained sections when compared to the saline-injected group (Fig. S6E and F). As determined by PAS staining, a trend toward an increased mucin area ratio was observed in the SG-BPCs-injected group compared to the saline-injected group (Fig. S6G and H). MTC staining demonstrated a decrease in the fibrotic area in the SG-BPCs-injected group compared to the saline-injected group, although this difference was not statistically significant (Fig. 6I and J). A tendency for increased *AQP5* expression was apparent in the SG-BPCs-injected compared to that in the saline-injected group (Fig. S6K). Restoration of salivary function in irradiated mouse models has confirmed its potential therapeutic effect in the treatment of SG dysfunction.

## Discussion

Our study demonstrated the successful application of a chemically reprogrammed 2D culture system for the expansion of SG-BPCs. By utilizing small-molecule cocktails, the need for feeder cells or extracellular matrices was effectively eliminated, simplifying the culture process while maintaining epithelial morphology and key progenitor cell markers such as KRT5, KRT19, and SOX9. Additionally, karyotyping results showed a normal chromatin structure in the presence of small-molecule compounds. Remarkably, these SG-BPCs could differentiate into various SG cell types, including acinar and myoepithelial cells. Furthermore, we observed that SG-BPCs have the potential to mitigate radiation-induced damage to mouse SG.

We examined different concentrations of the small-molecule compounds to determine the optimal CRC conditions. We identified the concentration that either promoted or suppressed cell growth. This suggests that precise concentration control is critical for the successful CRC when using small-molecule combinations. These results can be applied to similar cell culture experiments involving small-molecule compounds. Y-27632 facilitates proliferation by inhibiting apoptosis and enhances colony formation efficiency in serum-free media [48, 49]. A83-01 and LDN193189 inhibit the activity of several receptors [50, 51], including ACVR1B, TGF-β type I receptor kinase, ACVR1C, ACVR1, BMPR1A, and BMPR1B, which are known to play a role in epithelial-to-mesenchymal transition (EMT) [52, 53]. Notably, we found that SG cells cultured in A83-01 and LDN193189 for prolonged periods showed increased expression of *Vim*, an EMT marker. However, CDH1, an epithelial marker, was still expressed at the protein level. We speculated that basal progenitor cells partially express *Vim*, as they are cycling cells, but do not transition to EMT. Despite the upregulation of mRNA expression, the corresponding proteins were not expressed.

Previous studies have shown that myoepithelial cells are formed when the notch signal is disabled in SG organoid cultures [54, 55]. However, in this study, myoepithelial cells were discovered spontaneously during the 3D culture. It is believed that this occurred due to the reduction in the level of notch lateral activation as the cell–cell interaction decreased, which was caused by the very low concentration of Ca^2+^ ions in KEM. IF staining showed the upregulated expression of KRT7 in 2D cultures. To ensure temporal alignment between the 2D and 3D cultures, cells were cultured in 2D conditions for 9 d until they reached 90% confluence, facilitating contact inhibition and the initiation of differentiation [56]. The absence of KRT7 expression in the 3D culture is likely due to the suppression of KRT7 expression by TGF-βR inhibitors via a SMAD2/3-dependent mechanism [57].

The use of oncogenes such as hTert or conditional reprogramming culture with the NIH3T3 J2 feeder cell layer to immortalize cell lines is being phased out due to the risks of tumor development and unwanted cell incorporation during cell therapy. CRC using small-molecule compounds is relatively free of these issues [58]. Recent studies have reported that transient upregulation of Yamanaka factors induces cellular dedifferentiation and promotes rejuvenation, making them a promising approach in regenerative therapies [59, 60]. When SG epithelial cells were cultured in KEM, the expression of Yamanaka factors increased; however, the cells exhibited limited proliferation and reduced organoid formation efficiency. Interestingly, small molecules of YAL reduced *SOX2* and *POU5F1* expression in SG-BPC cultures. Co-expression of *SOX2* and *POU5F1* induces cell transformation by increasing the pluripotency in oral mucosa [61]. This suggests that small molecules inhibit cell transformation. Flow cytometric analysis revealed that SG-BPCs exhibited phenotypic characteristics of basal cells, suggesting their potential as a basal cell population.

Normal cells have various regulatory mechanisms that prevent tumorigenesis, one of which involves the activation of cellular senescence pathways upon oncogene activation [62]. We confirmed that these small molecules inhibit cellular senescence and apoptosis in SG-BPCs. Senescence-associated anti-apoptotic pathways (SCAPs) allow senescent cells to resist apoptosis, with increased expression of anti-apoptotic proteins such as BCL-xL playing a significant role. SCAPs selectively induce apoptosis in senescent cells under specific conditions [63]​. BCL-xL and BMF, as members of the BCL-2 family, are critical for modulating cell death during senescence progression, making them important therapeutic targets for inducing apoptosis [64]. Additionally, we performed cytokine array analysis to identify the growth factors and cytokines secreted by SG-BPCs. IL-6 may contribute to the stemness of stem cells. Increased IL-6 levels activate cell proliferation and maintain the undifferentiated state [65–67]. In addition, IL-33 has been reported to play a role in epithelial regeneration and stem cell proliferation [68, 69]. IL-33 regulates epithelial regeneration by inducing the production of epidermal growth factors (EGF) after radiation injury in vivo and increases stem cell proliferation *in vitro* [70]. The CXCL10 regulates self-renewal and differentiation of stem cells, and CXCL9 increases proliferation and homing effects but does not affect stem cell differentiation [71]. Furthermore, the overexpression of CXCL9 in stem cells showed a higher anti-fibrotic effect after injury *in vivo* [72].

Transcriptome analysis of KEM- and KEM + YAL-cultured cells was performed to elucidate the mechanisms underlying the effects of small-molecule compounds. Treatment with small-molecule compounds enhanced the activation of the NF-κB signaling pathway. NF-κB is a key regulator of cell survival, protecting cells from apoptosis induced by various stress stimuli [73]. When human bronchial epithelial cells were treated with an NF-κB inhibitor or NF-κB expression was knocked down using siRNA, an increase in DNA damage-induced cell death was observed. This indicates that NF-κB activation upregulates anti-apoptotic markers, thereby promoting cell survival [74]. Therefore, it appears that the small-molecule compounds enhance the survival of SG-BPCs by activating the NF-κB signaling pathway.

We investigated the therapeutic efficacy of SG-BPCs in an irradiated mouse model. The SG-BPCs-treated group showed enhanced SG function, evidenced by increased saliva production at 3 and 8 weeks after SG-BPCs injection. We observed an increase in the expression of AQP5, a marker of acinar cells, in the SG-BPCs-injected group, along with functional improvement. Additionally, we observed an increase in SG weight at 8 weeks, but no significant difference at 3 weeks, indicating the long-term efficacy of SG-BPCs after SG-BPCs injection. Moreover, the levels of markers indicative of cell death and senescence were significantly lower in this group. These results suggest that injected SG-BPCs exert a cytoprotective effect through paracrine mechanisms, probably involving the secretion of growth factors and cytokines.

Further detailed comparative studies, such as whole-genome analyses, are required before clinical translation. It is necessary to identify the distinct subtype of progenitor cells expanded by CRC through single-cell RNA sequencing analysis. To validate the potential for future clinical applications, further preclinical assessments of safety and tumorigenicity of SG-BPCs are necessary. Animal experiments must be conducted to verify the engraftment, differentiation, and residual efficiencies within actual tissues. In addition, clinical research is required to determine the efficiency and mode of action of chemically reprogrammed SG-BPCs in SG hypofunctional diseases.

The proposed approach offers several key advantages. First, it significantly simplifies the culture process and reduces the time and labor required to maintain the SG-BPCs. Second, it minimizes the risk of contamination and variability associated with feeder cells, ensuring greater reproducibility and reliability of the experimental outcomes. Third, it promotes the robust proliferation and long-term maintenance of SG-BPCs, providing a valuable resource for studying SG biology and developing regenerative therapies. Furthermore, it holds great promise for advancing regenerative medicine applications such as tissue engineering and cell therapy for SG disorders. By addressing the limitations of the previous culture methods, this study provides an efficient and reliable platform for studying SG-BPCs.

## Conclusion

We successfully chemically reprogrammed and expanded SG-BPCs. Although cell-based therapy using MSCs can help maintain the stem cell niche, the healing effect may be limited when the stem cell pool itself is damaged by radiation exposure or a poor microenvironment in the tissue. Therefore, we propose that the chemically reprogrammed SG-BPCs isolated using our CRC method can replenish the stem cell pool and differentiate into SG cells. We verified that these chemically reprogrammed cells have basal-like epithelial characteristics and stem-like properties and can differentiate into SG cells. Additionally, we confirmed the therapeutic efficacy of SG-BPCs in an irradiated mouse model.

## Supplementary Information

Supplementary material 1.

Supplementary material 2.

Supplementary material 3.

Supplementary material 4.

## Data Availability

All additional files are included in the manuscript. All supporting data can be inquired to the corresponding authors upon reasonable request.

## Abbreviations

*SG*: Salivary gland
*CRC*: Chemical reprogramming culture
*SG-BPCs*: SG-basal progenitor cells
*PD*: Population doubling level
*MSCs*: Mesenchymal stem cells
*SG-EpSPCs*: SG-resident epithelial stem or progenitor cells
*2D*: Two-dimensional
*3D*: Three-dimensional
*TGF*: Transforming growth factor
*BMP*: Bone morphogenetic protein
*iPSC*: Induced pluripotent stem cells
*KEM*: Keratinocyte expansion medium
*PBS*: Dulbecco’s phosphate-buffered saline
*PDT*: Population doubling time
*YAL*: Y-27632, A83-01, and LDN193189
*ICC*: Immunocytochemistry staining
*qRT-PCR*: Quantitative real time-PCR
*β-gal*: β-Galactosidase
*TUNEL*: Terminal deoxynucleotidyl transferase dUTP nick end labeling
*SMG*: Submandibular salivary gland
*HE*: Hematoxylin and eosin
*PAS*: Periodic acid–schiff
*MTC*: Masson’s trichrome
*ANOVA*: One-way analysis of variance
*ROCK*: Rho kinase
*EGF*: Epidermal growth factor
*CALB2*: Calbindin 2
*ADAM28*: A disintegrin and metalloproteinase
*ICAM1*: Intercellular adhesion molecule 1
*MYB*: Myeloblastosis oncogene
*TAGLN*: Transgelin
*ID3*: Inhibitor of DNA binding 3
*EMT*: Epithelial-to-mesenchymal transition
*SCAPs*: Senescence-associated anti-apoptotic pathways

## Declarations

### Artificial intelligence

The authors declare that artificial intelligence-generated work was not used in this manuscript.
