# Supplementary material for: Chemical reprogramming culture for the expansion of salivary gland epithelial basal progenitor cells
Source: Stem Cell Res Ther. 2025 Apr 18;16:187. doi: 10.1186/s13287-025-04295-5 (PMC12008940; doi:10.1186/s13287-025-04295-5)
Supplement: Supplementary file 1 — Supplementary material 1. [file 13287_2025_4295_MOESM1_ESM.docx]

**Supplementary figures**

**
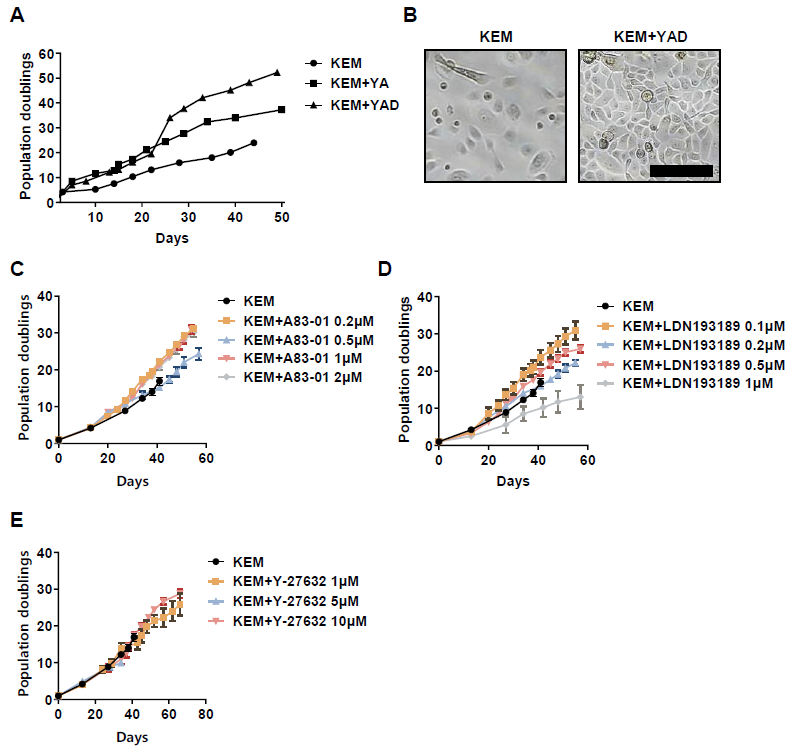
**

**Fig. S1 Optimal condition of TGF-β, BMP, and ROCK inhibitors support SG epithelial cell** **proliferation**

(A) Population doubling of SG epithelial cells cultured in KEM in the absence and presence of small-molecule combinations. (B) Representative images of SG epithelial cells cultured in the absence and presence of small-molecule cocktails. Scale bar: 100 μm. We used KSFM as the culture medium for both experiments (A) and (B). (C), (D), and (E) Optimal concentration of TGF-β, BMP, and ROCK inhibitors promote SG epithelial cell proliferation.


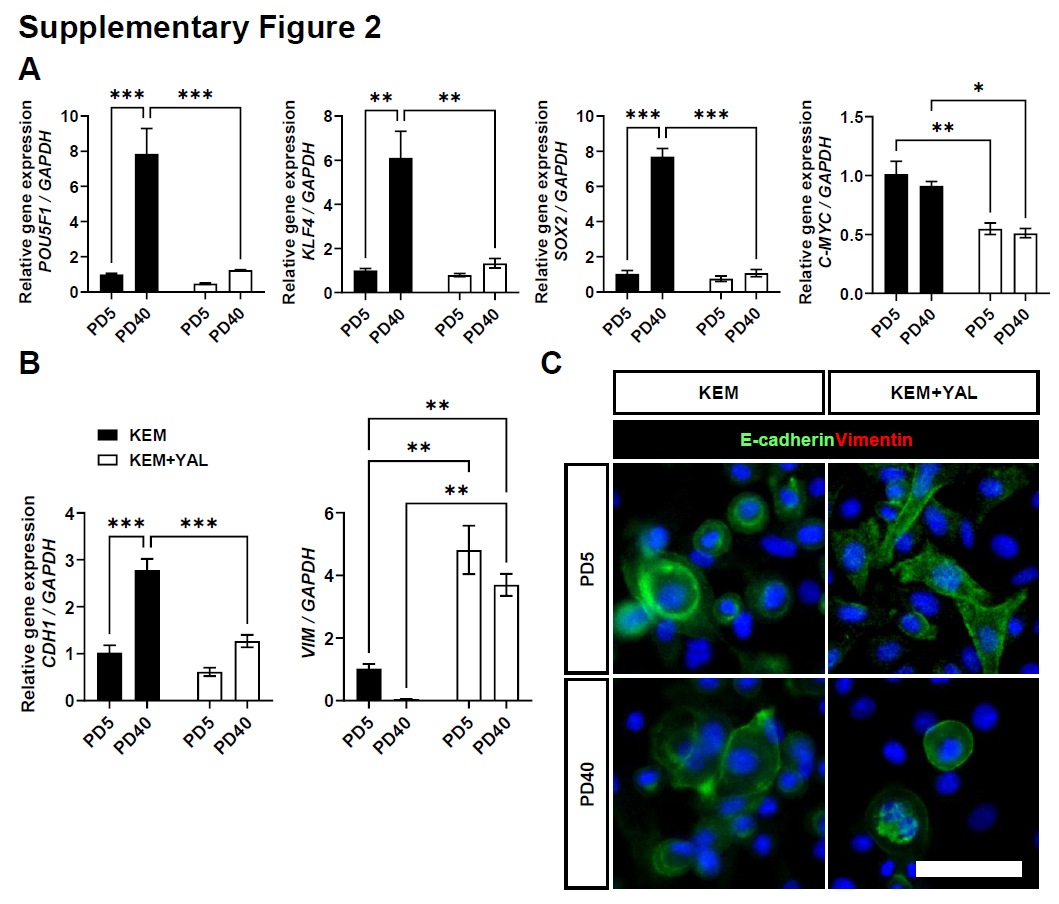


**Fig. S2 Comparison of cellular properties in cells cultured under KEM and KEM+YAL conditions**

(A) Gene expression of Yamanaka factor in both small molecules untreated and treated SG epithelial cells. (B) mRNA expression of epithelial cell marker (*CDH1*) and transit-amplifying cell marker (*VIM*). (C) ICC staining for E-Cadherin (epithelial cell, green) and Vimentin (transit−amplifying cell, red). Scale bar: 50 μm. The data are representative of three independent experiments performed in triplicate and are expressed as mean ± SEM. * = *p* < 0.05, ** = *p* < 0.01, *** = *p* < 0.001.

**
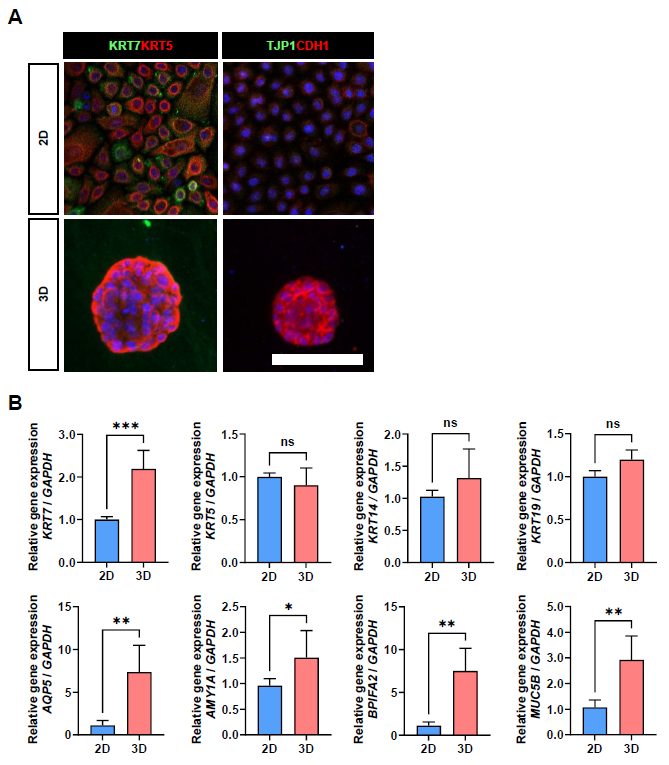
**

**Fig. S3** **Differentiation potential of cells cultured under KEM+YAL conditions**

(A) Immunofluorescence microscopy of KRT7, KRT5, TJP1, and CDH1 on both 2D and 3D cultures for 9 days**.** Scale bar: 100 μm. (B) The expressions of *KRT7, KRT5, KRT14, KRT19, AQP5, AMY1A, BPIFA2*, and *MUC5B* between 2D and 3D cultured cells, are determined by qRT-PCR. Data are representative of at least three independent experiments and are presented as mean ± SD. Welch Two Sample t-test (alpha = 0.05) is conducted on data presented. ns = not significant, * = *p* < 0.05, ** = *p* < 0.01, *** = *p* < 0.001.


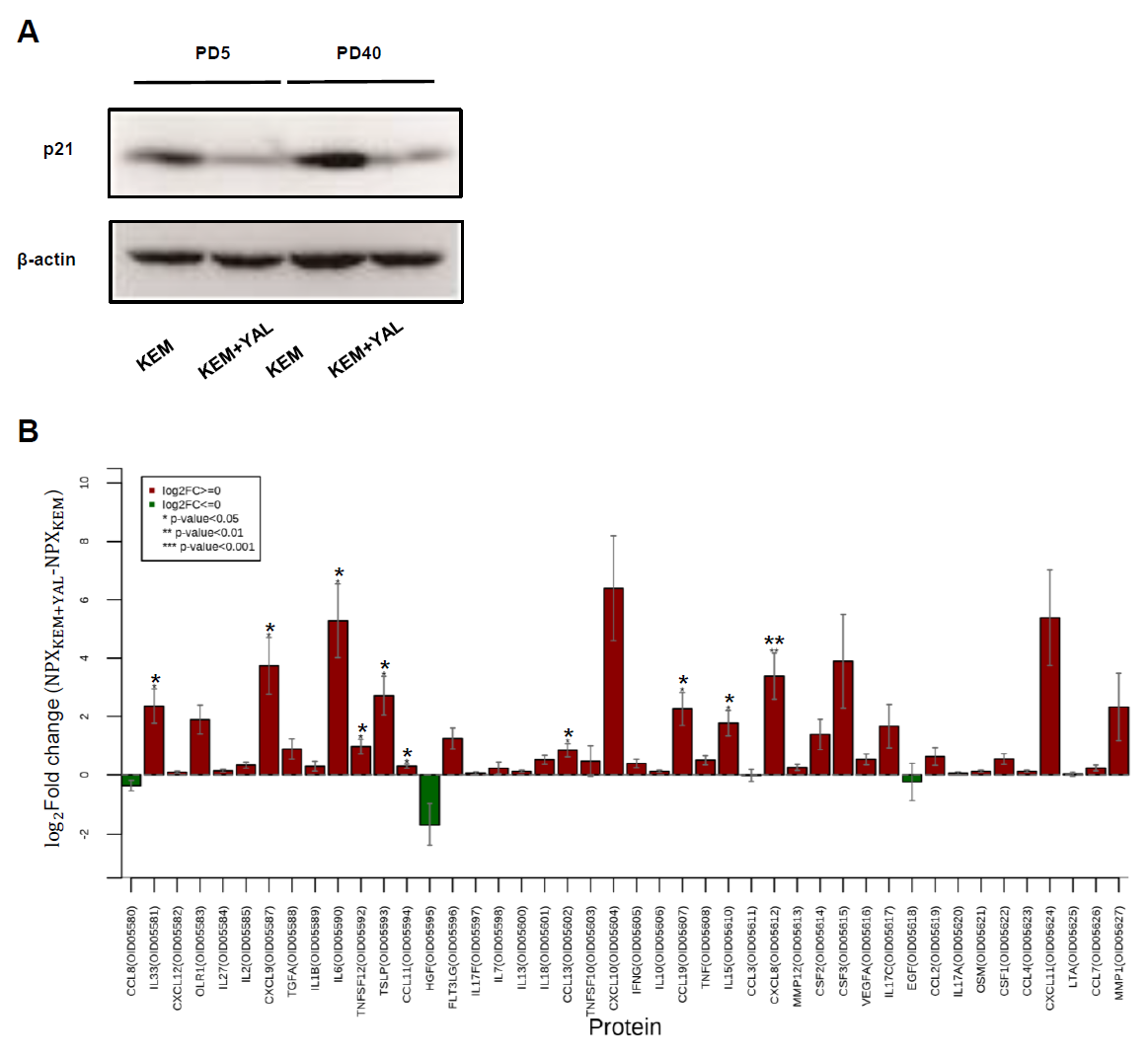
**Fig. S4 Protein expression profiles in cells cultured under KEM and KEM+YAL conditions**

(A) Western blotting is performed to analyze protein expression levels of p21 and β-actin in PD5 and PD40 cells cultured without or with small-molecule cocktails. Full-length blots are presented in Fig. S7B. (B) Fold change plot for 48 cytokines indicating the relative protein expression in PD40 cells cultured without or with small-molecule cocktails. Bars are colored in (Red [Up regulated] / Green [Down regulated]) depending on the fold change. Welch Two Sample t-test (alpha = 0.05) is conducted on the data presented. * = *p* < 0.05, ** = *p* < 0.01.


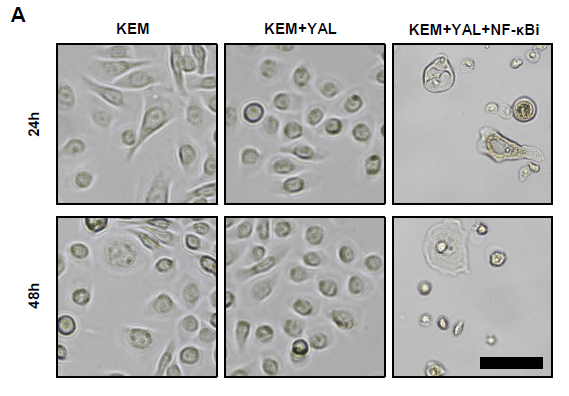


**Fig. S5 Inhibition of NF-κB signaling suppresses proliferation of SG-BPCs**

(A) Representative images of the cells cultured under the KEM, KEM+YAL, and KEM+YAL+NF-κBi conditions for 24 and 48 h at PD40 time-point. Scale bar: 100 μm.


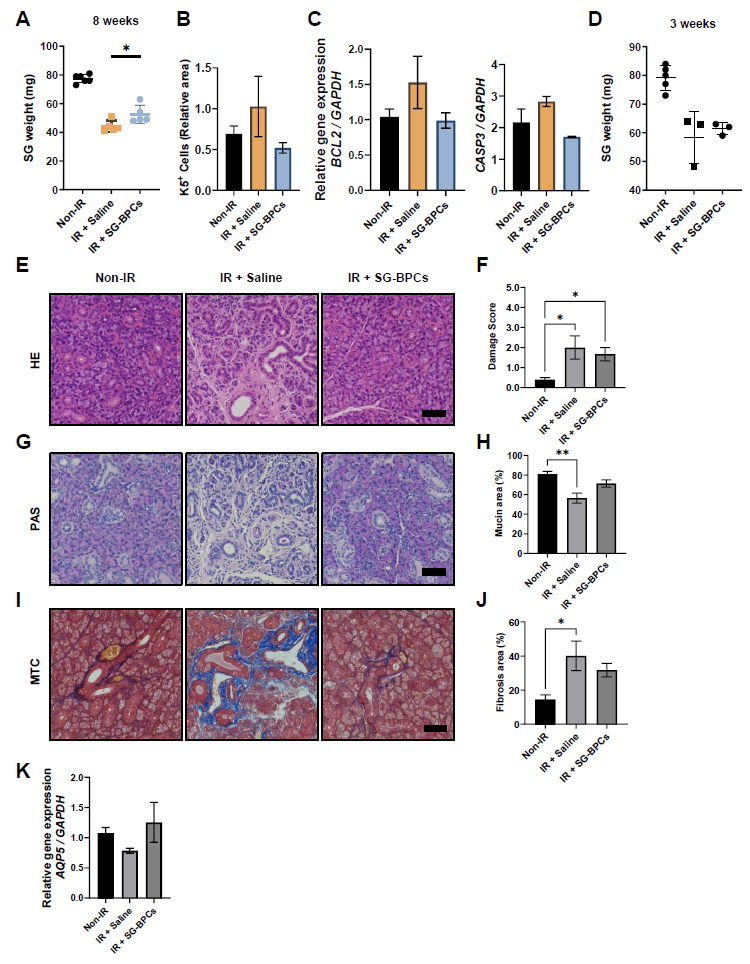


**Fig. S6 Therapeutic efficacy of SG-BPCs in a mouse radiation model**

(A) SG weight measured at 8 weeks after SG-BPCs injection. (B) Quantification of KRT5 cells for Immunofluorescence staining at 8 weeks after SG-BPCs injection. (C) Gene expression analysis of markers associated with cellular apoptosis (*BCL2* and *CASP3*). (D) SG weight was measured 3 weeks after SG-BPCs injection. Two-way ANOVA (alpha = 0.05) is conducted on data presented in (A) and (D), followed by Tukey’s multiple comparisons. The data are representative of three independent experiments performed in triplicate and are expressed as mean $\pm$SEM. * = *p* < 0.05. (E), (G), and (I) Representative histological images of HE, PAS, and MTC staining 3 weeks after SG-BPCs injection, respectively. Scale bar: 50 μm. (F), (H), and (J) Quantification of SG damage score, ratio of mucin area, and fibrosis area, respectively. Two-way ANOVA (alpha = 0.05) is conducted on data presented in (F), (H), and (J) followed by Tukey’s multiple comparisons. The data are representative of three independent experiments performed in triplicate and are expressed as mean $\pm$SEM. * = *p* < 0.05, ** = *p* < 0.01. (K) Gene expression analysis of marker associated with acinar cell (*AQP5*).
