# Supplementary material for: Chemical reprogramming culture for the expansion of salivary gland epithelial basal progenitor cells
Source: Stem Cell Res Ther. 2025 Apr 18;16:187. doi: 10.1186/s13287-025-04295-5 (PMC12008940; doi:10.1186/s13287-025-04295-5)
Supplement: Supplementary file 2 — Supplementary material 2. [file 13287_2025_4295_MOESM2_ESM.pdf]

Supplementary Figure 7

A

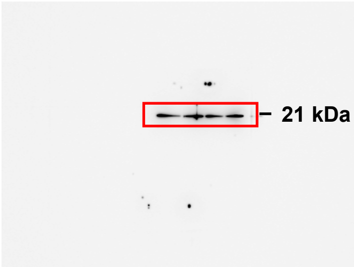

Figure 2D-Sox2

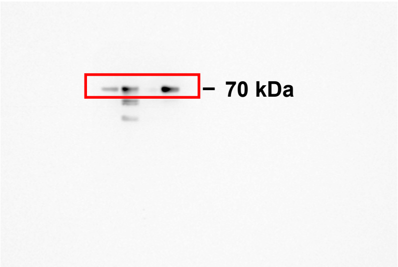

Figure 2D-Sox9

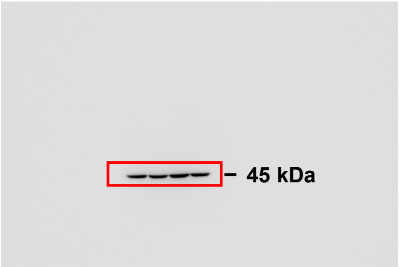

Figure 2D-β-actin

B

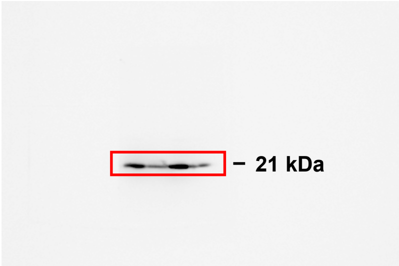

Supplementary Figure 4A-p21

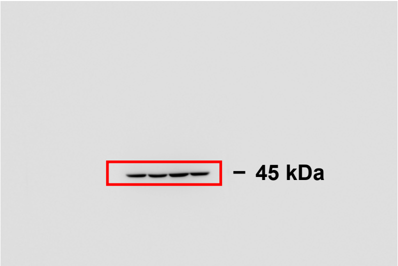

Supplementary Figure 4A-β-actin

C

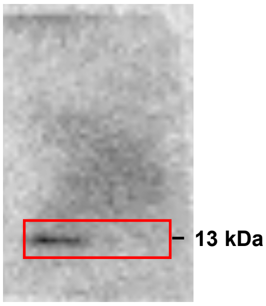

Figure 5C-ID3

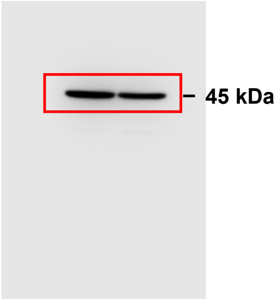

Figure 5C-β-actin

D

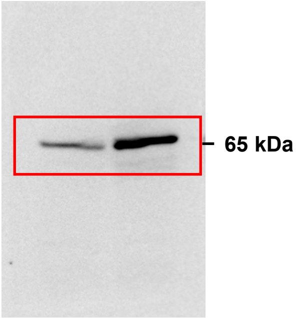

Figure 5E-p-p65

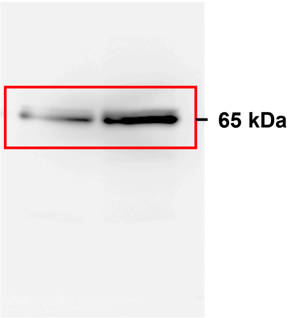

Figure 5E-p65

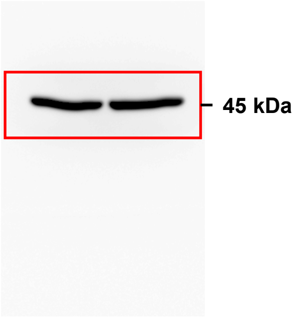

Figure 5E- β-actin
