# Supplementary material for: Chemical reprogramming culture for the expansion of salivary gland epithelial basal progenitor cells
Source: Stem Cell Res Ther. 2025 Apr 18;16:187. doi: 10.1186/s13287-025-04295-5 (PMC12008940; doi:10.1186/s13287-025-04295-5)
Supplement: Supplementary file 4 — Supplementary material 4. [file 13287_2025_4295_MOESM4_ESM.docx]

**Supplementary Table 1. Primer list for Quantitative RT-PCR**

| **Human genes** | **Forward sequence (5′-3′)** | **Reverse sequence (5′-3′)** |
| --- | --- | --- |
| CDKN2A | CTC GTG CTG ATG CTA CTG AGG A | GGT CCG CGC AGT TGG GCT CC |
| CDKN1A | AGG TGG ACC TGG AGA CTC TCA G | TCC TCT TGG AGA AGA TCA GCC G |
| TENM2 | CCT CTC GAA ATG TGA CCA GCA TC | GCG GTA GAT TCT CCT GCT GTT G |
| POU5F1 | GGT GGA GGA AGC TGA CAA CA | CAC GAG GGT TTC TGC TTT GC |
| SOX2 | GCT ACA GCA TGA TGC AGG ACC A | TCT GCG AGC TGG TCA TGG AGT T |
| SOX9 | GAC TTC TGA ACG AGA GCG AGA | CCG TTC TTC ACC GAC TTC CTC |
| KLF4 | CAT CTC AAG GCA CAC CTG CGA A | TCG GTC GCA TTT TTG GCA CTG G |
| KRT19 | AGC TAG AGG TGA AGA TCC GCG A | GCA GGA CAA TCC TGG AGT TCT C |
| VIM | AGG CAA AGC AGG AGT CCA CTG A | ATC TGG CGT TCC AGG GAC TCA T |
| KRT5 | GCT GCC TAC ATG AAC AAG GTG G | ATG GAG AGG ACC ACT GAG GTG T |
| KRT7 | TGT GGA TGC TGC CTA CAT GAG C | AGC ACC ACA GAT GTG TCG GAG A |
| AQP5 | TAC GGT GTG GCA CCG CTC AAT G | AGT CAG TGG AGG CGA AGA TGC A |
| c-MYC | CCT GGT GCT CCA TGA GGA GAC | CAG ACT CTG ACC TTT TGC CAG G |
| CDH1 | GCC TCC TGA AAA GAG AGT GGA AG | TGG CAG TGT CTC TCC AAA TCC G |
| KRT14 | TGC CGA GGA ATG GTT CTT CAC C | GCA GCT CAA TCT CCA GGT TCT G |
| AMY1A | GAT AAT GGG AGC AAC CAA GTG GC | CAG TAT GTG CCA GCA GGA AGA C |
| BPIFA2 | CCT GGA TGT CAA AGC TGA ACC G | AGG TCC AAG GAG GCT TTC AGG T |
| MUC5B | CTG CTA CGA CAA GGA CGG AAA C | AAG GCT GTG AGC GCA CTG GAT G |
| MMP10 | TCC AGG CTG TAT GAA GGA GAG G | GGT AGG CAT GAG CCA AAC TGT G |
| BMF | CAG TGG CAA CAT CAA GCA GAG G | GCA AGG TTG TGC AGG AAG AGG A |
| BCL-xL | GCC ACT TAC CTG AAT GAC CAC C | AAC CAG CGG TTG AAG CGT TCC T |
| ADAM28 | GTA CTG TCG CAG AGT GGA TGA C | GTC ACT ATC CGT CCT TTC CAG G |
| ID3 | CAG CTT AGC CAG GTG GAA ATC C | GTC GTT GGA GAT GAC AAG TTC CG |
| TAGLN | TCC AGG TCT GGC TGA AGA ATG G | CTG CTC CAT CTG CTT GAA GAC C |
| MYB | GGG AAC AGA TGG GCA GAA ATC G | GCT GGC TTT TGA AGA CTC CTG C |
| GAPDH | GTC TCC TCT GAC TTC AAC AGC G | ACC ACC CTG TTG CTG TAG CCA A |

| **Mouse genes** | **Forward sequence (5′-3′)** | **Reverse sequence (5′-3′)** |
| --- | --- | --- |
| CDKN1A | TCG CTG TCT TGC ACT CTG GTG T | CCA ATC TGC GCT TGG AGT GAT AG |
| AQP5 | CAA CAC AAC ACC AGG CAA GG | AGT GTG ACC GAC AAG CCA AT |
| FAS | CTG CGA TTC TCC TGG CTG TGA A | CAA CAA CCA TAG GCG ATT TCT GG |
| BAX | CCC ACC AGC TCT GAA CAG AT | CTC CAA GGT CAG CTC AGG TG |
| BCL2 | GCG TCA ACA GGG AGA TGT CA | CAT CCC AGC CTC CGT TAT CC |
| CASP3 | GGA GTC TGA CTG GAA AGC CGA A | CTT CTG GCA AGC CAT CTC CTC A |
| GAPDH | CAT CAC TGC CAC CCA GAA GAC TG | ATG CCA GTG AGC TTC CCG TTC AG |

**Supplementary Table 2. Antibody list for Flow cytometry analysis.**

| **Antigen** | **Fluorochrome** | **Company** | **Cat#** |
| --- | --- | --- | --- |
| HLA-DR | APC/Cyanine7 | BioLegend | 307617 |
| CD31 | FITC | BioLegend | 303104 |
| CD34 | Alexa Fluor 700 | BioLegend | 343621 |
| CD90 | APC/Cyanine7 | BioLegend | 328131 |
| CD117 | PerCP/Cyanine5.5 | BioLegend | 313213 |
| CD29 | PE/Dazzle 594 | BioLegend | 303031 |
| CD44 | Brilliant Violet 785 | BioLegend | 103059 |
| CD166 | PerCP/Cyanine5.5 | BioLegend | 343907 |
| CD146 | PE/Cyanine7 | BioLegend | 361007 |
| CD26 | PE | BioLegend | 302705 |
| CD49f | PE/Cyanine7 | BioLegend | 313622 |
|  | PerCP/Cyanine5.5 | BioLegend | 400149 |
|  | APC/Cyanine7 | BioLegend | 400127 |
|  | PE/Dazzle™ 594 | BioLegend | 400175 |
|  | PE/Cyanine7 | BioLegend | 400125 |
|  | Alexa Fluor 700 | BioLegend | 400247 |
|  | Brilliant Violet 785 | BioLegend | 400647 |
|  | PE | BioLegend | 400211 |
|  | FITC | BioLegend | 400107 |
|  | PE/Cyanine7 | BioLegend | 400521 |
